# Continuous Rhythm Monitoring and Signal-Averaged Electrocardiogram to Risk Stratify Ventricular Arrhythmia in Mitral Valve Prolapse

**DOI:** 10.1016/j.jacadv.2025.101643

**Published:** 2025-03-19

**Authors:** Chee Loong Chow, Hui-Chen Han, Andrew W. Teh, Ruth P. Lim, Hamid Salehi, Anoop N. Koshy, David L. Hare, Prashanthan Sanders, Omar Farouque, Han S. Lim

**Affiliations:** a Department of Cardiology, Northern Health and University of Melbourne, Melbourne, Victoria, Australia; b Department of Cardiology, Austin Health and University of Melbourne, Melbourne, Victoria, Australia; c Department of Cardiology, Eastern Health and Monash University, Melbourne, Victoria, Australia; d Department of Radiology, Austin Health and University of Melbourne, Melbourne, Victoria, Australia; e Centre for Heart Rhythm Disorders, University of Adelaide and Royal Adelaide Hospital, Adelaide, South Australia, Australia

**Keywords:** implantable loop recorder, mitral valve prolapse, signal-averaged electrocardiogram

Mitral valve prolapse (MVP) is associated with sudden cardiac death (SCD), with autopsy studies finding MVP in up to 7% of SCD.^1^ Arrhythmic MVP—a term used to characterize a phenotype at risk of life-threatening arrhythmia—is frequently associated with risk markers such as bileaflet MVP, polymorphic premature ventricular contractions, mitral annular disjunction, and presence of myocardial fibrosis. The incidence of ventricular arrhythmia can be as high as 4% per person-year in patients with MVP and mitral annular disjunction.^2^ We prospectively studied the incidence of ventricular arrhythmia events in patients with MVP with continuous rhythm monitoring using implantable loop recorder (ILR). Additionally, the role of signal-averaged electrocardiogram (SAECG) and cardiac magnetic resonance imaging in risk stratification were also examined.

## Methods

All patients with echocardiography diagnosis of MVP (defined as atrial displacement ≥2 mm of the mitral valve during systole) at the Austin Hospital, Melbourne, Australia, between May 2016 and May 2019 were invited to participate. Exclusion criteria were age <18 or >85 years or those with an existing cardiovascular implantable electronic device. Recruited patients underwent ILR implantation, prospective cardiac assessment with baseline ECG, Holter monitor, SAECG, and cardiac magnetic resonance imaging to investigate for the presence of late gadolinium enhancement (LGE). Ethical approval for this study was granted by Austin Health Human Research Ethics Committee.

The outcome assessed was a composite of: 1) ventricular fibrillation; 2) sustained ventricular tachycardia (VT) (consecutive ventricular beats >120 beats/min for >30 seconds OR if <30 seconds, associated with symptoms of hemodynamic compromise such as syncope); and 3) nsonsustained VT (NSVT) of >8 consecutive beats at >179 beats/min, consistent with common definitions of “severe arrhythmia” in the literature^2^^,^^3^ associated excess mortality.^3^ Ventricular arrhythmia episodes must not have alternative etiologies, for instance flow-significant coronary artery disease. Incidence rate is presented for time-dependent primary outcome, and Cox regression analysis performed to assess for patient characteristics and factors associated with event outcome.

## Results

Twenty-two MVP patients were recruited, with a mean follow-up duration of 677.5 days (range: 87-891 days). Thirteen (59.1%) patients were female, with an average age of 64 years (range: 43-75 years). Most patients had bileaflet MVP (n = 19, 86.4%).

Six (27.3%) patients met the primary composite endpoint, with most (5 of 6 patients) due to NSVT >8 consecutive beats at >179 bpm, and one due to sustained VT (>30 seconds) associated with presyncope. This equates to an incidence rate of 14.7 events per 100 person-years. The mean time to VA detection post-ILR implant was 352 days (range: 113-698 days). When compared to their screening 24-hour Holter where no severe arrhythmia was detected, the number-needed-to-treat (in this case the number of ILR implantation needed to detect an event of severe arrhythmia) was 3.7. Of these 6 patients, one required an implantable cardiac defibrillator insertion and anti-arrhythmic commencement, 3 required anti-arrhythmic commencement or uptitration, one was maintained on usual beta-blocker (bisoprolol 1.25 mg unchanged), and one did not have any active intervention as he only had an asymptomatic short run.

Several risk factors were examined for potential association with the primary composite endpoint of severe VA (Table 1). Only SAECG, specifically filtered QRS duration of >120 ms was associated with the primary outcome (HR: 12.06; 95% CI: 1.34-108.57; *P* = 0.03). The other components of SAECG were not associated with VA incidence, such as low amplitude signal duration >38 ms (*P* = 0.57) and root mean square of amplitude in the terminal 40 ms of <20 uV (*P* = 0.51). LGE did not achieve a statistically significant association with the primary composite outcome (HR: 5.07; 95% CI: 0.59-43.87; *P* = 0.14). However, anterior mitral leaflet displacement demonstrated a trend toward statistical significance (HR: 0.63; 95% CI: 0.40-1.00; *P* = 0.05).

**Table 1:** Characteristics of MVP Patients With and Without VA

	VA	No VA	HR (95% CI)	*P* Value
	(n = 6)	(n = 16)		
Follow-up duration (d)	717 (488-891)	663 (87-886)	1.00 (0.99-1.01)	0.77
Age (y)	59.2 (46-71)	66.3 (43-75)	0.94 (0.86-1.02)	0.12
Female	2 (33.3%)	11 (68.8%)	5.20 (0.91-29.71)	0.06
Clinical characteristics				
Palpitations	4 (66.8%)	11 (68.8%)	1.02 (0.19-5.60)	0.98
Syncope	2 (33.3%)	5 (31.3%)	0.98 (0.17-5.46)	0.98
History of atrial fibrillation	1 (16.7%)	1 (6.3%)	1.92 (0.22-16.89)	0.56
Anti-arrhythmic				
Beta-blockers	1 (16.7%)	5 (31.3%)	0.40 (0.05-3.46)	0.40
Calcium-channel blockers	0	1 (6.3%)	0.05 (0.00- >100)	0.75
Amiodarone	0	1 (6.3%)	0.04 (0.00- >100)	0.64
ECG				
QRS duration (ms)	104.2 (80-138)	96.3 (76-120)	1.05 (0.98-1.13)	0.17
Inferior-lateral TWI	2 (33.3%)	4 (25.0%)	0.99 (0.18-5.46)	0.99
Holter monitor				
PVC %	3.2 (0.0-10.5)	5.0 (0.0-24.6)	0.96 (0.83-1.11)	0.59
PVC/Hour	181.7 (0.0-372.3)	214.0 (0.9-1,035.7)	1.00 (0.99-1.00)	0.62
Presence of ventricular couplets^a^	5 (83.3%)	13 (81.3%)	1.06 (0.12-9.48)	0.96
Presence of ventricular triplets^a^	4 (66.8%)	8 (50.0%)	3.30 (0.37-29.57)	0.29
Echocardiogram				
LVEF (%)	62.3 (55-72)	60.2 (50-75)	1.03 (0.90-1.18)	0.68
LVEDD (mm)	53.7 (52-55)	53.2 (44-63)	1.03 (0.87-1.21)	0.76
Presence of MAD	1 (16.7%)	3 (18.8%)	1.34 (0.15-11.63)	0.79
MAD distance (mm)	9.4 (9.4)	8.1 (3.7-11.8)	1.14 (0.52-2.49)	0.75
Anterior leaflet length (mm)	26.4 (19.9-31.6)	29.0 (21.4-39.2)	0.93 (0.77-1.13)	0.47
Anterior leaflet thickness (mm)	5.7 (3.5-7.2)	6.3 (4.2-9.8)	0.70 (0.30-1.60)	0.39
Anterior leaflet displacement (mm)	4.1 (0.0-5.8)	5.8 (2.3-9.6)	0.63 (0.40-1.00)	0.05
Anterior leaflet area (mm^2^)	94.8 (60-133)	118.9 (76-189)	0.97 (0.94-1.01)	0.14
Posterior leaflet length (mm)	19.4 (16.7-24.1)	19.3 (11.4-30.0)	1.04 (0.84-1.28)	0.74
Posterior leaflet thickness (mm)	5.8 (5.4-6.8)	5.9 (2.2-9.3)	0.93 (0.53-1.64)	0.79
Posterior leaflet displacement (mm)	4.7 (2.5-7.4)	6.1 (0.0-12.3)	0.83 (0.61-1.14)	0.25
Posterior leaflet area (mm^2^)	73.4 (43-124)	81.3 (33-148)	1.00 (0.97-1.02)	0.78
Presence of bileaflet prolapse	5 (83.3%)	14 (87.5%)	0.25 (0.03-2.47)	0.24
Presence of moderate or severe MR	4 (66.8%)	8 (50.0%)	1.91 (0.34-10.73)	0.46
Cardiac magnetic resonance				
Presence of LGE	5 (83.3%)	5 (35.7%)	5.07 (0.59-43.87)	0.14
SAECG				
Filtered QRSd >120 ms	4 (80.0%)	2 (12.5%)	12.06 (1.34-108.57)	**0.03**
HFLA duration >38 ms	3 (60.0%)	6 (37.5%)	1.67 (0.28-10.03)	0.57
RMS40 <20 uV	3 (60.0%)	6 (37.5%)	1.82 (0.30-10.91)	0.51

Values are median (range) or n (%) unless otherwise indicated. **Bold** signifies significant *P* value of <0.05.

ECG = electrocardiogram; HFLA = high frequency low amplitude (<40 uV); LGE = late gadolinium enhancement; LVEDD = left ventricular end diastolic diameter; LVEF = left ventricular ejection fraction; MAD = mitral annular disjunction; MR = mitral regurgitation; MVP = mitral valve prolapse; PVC = premature ventricular contraction; QRSd = QRS duration; RMS40 = root mean square signal amplitude of the last 40 ms of the signal; SAECG = signal-averaged ECG; TWI = T-wave inversion; VA = severe ventricular arrhythmia as defined in-manuscript.

a Presence of couplets includes any ventricular runs longer than 2 consecutive beats; presence of triplets includes only ventricular runs with longer than 3 consecutive beats. No patients had severe VA (as defined in-manuscript) on Holter monitor.

## Discussion

This observational work is important for several key findings. Firstly, the incidence of severe VA is high (27.3%) in this cohort largely represented by patients with MVP and bileaflet redundancy (86.4%). The mean time to VA detection was 352 days post-ILR implant. The current European Heart Rhythm Association expert consensus statement recommends using periodic Holter to screen asymptomatic MVP patients,^4^ but this could underestimate the burden of severe arrhythmia that confers excess mortality risk. This observation argues for the role of longer continuous cardiac monitoring to identify patients with arrhythmic phenotypes with greater sensitivity. The large representation of bileaflet redundancy in this cohort also represents a limitation, as it does not represent the general MVP cohort at large.

Secondly, filtered QRS duration >120 ms on SAECG was associated with primary outcome of severe arrhythmia. Currently, SAECG is not used in routine clinical practice to predict the risk of arrhythmic events in patients with MVP. Our study presents a novel perspective on the potential of SAECG being an adjunct tool in the risk stratification of patients. Further work is required to ascertain the sensitivity, specificity, positive and negative predictive values of SAECG to guide its utility.

In addition, left ventricular fibrosis in patients with MVP has been shown to be associated with SCD in previous studies,^1^^,^^5^ and LGE has been correlated with NSVT of ≥3 beats.^1^ In our cohort, the presence of LGE did not achieve statistical significance. This is most likely due to the limitation of a small cohort size for this study and could be an important topic for further study.

In conclusion, our study demonstrates a potential role for continuous cardiac monitoring for risk stratification of VAs in patients with MVP, especially in the setting of bileaflet MV prolapse. In addition, the usefulness of SAECG in risk stratification of arrhythmic risk deserves further study.

## Funding support and author disclosures

Drs Han and H.S. Lim have received funding from Austin Medical Research Foundation (grant number 2-1252); and research materials from Abbott. Both Austin Medical Research Foundation and Abbott were not involved with the study design, data collection, and analysis or manuscript preparation. Dr Han has received research materials from Abbott. Dr Sanders has served on the advisory board of Boston Scientific, Medtronic, CathRx, Pacemate, and Abbott; and the University of Adelaide has received on his behalf research funding from Medtronic, Abbott, Boston Scientific, and Becton Dickinson. Dr H.S. Lim has received research support from Abbott and Medtronic. All other authors have reported that they have no relationships relevant to the contents of this paper to disclose.
